# Intravenous Mistletoe Treatment in Integrative Cancer Care: A Qualitative Study Exploring the Procedures, Concepts, and Observations of Expert Doctors

**DOI:** 10.1155/2016/4628287

**Published:** 2016-04-24

**Authors:** Gunver S. Kienle, Milena Mussler, Dieter Fuchs, Helmut Kiene

**Affiliations:** ^1^Institute for Applied Epistemology and Medical Methodology, University of Witten/Herdecke, Zechenweg 6, 79111 Freiburg, Germany; ^2^Center for Complementary Medicine, Institute for Environmental Health Sciences and Hospital Infection Control, University Medical Center Freiburg, Breisacher Strasse 115B, 79106 Freiburg, Germany; ^3^Department of Theology, Caritas Sciences, University of Freiburg, Werthmannplatz 3, 79098 Freiburg, Germany

## Abstract

*Background*. Mistletoe therapy (MT) is widely used in patient-centered integrative cancer care. The objective of this study was to explore the concepts, procedures, and observations of expert doctors, with a focus on intravenous MT.* Method*. A qualitative interview study was conducted with 35 highly experienced doctors specialized in integrative and anthroposophic medicine. Structured qualitative content analysis was applied. For triangulation, the results were compared with external evidence that was systematically collected, reviewed, and presented.* Results*. Doctors perform individualized patient assessments that lead to multimodal treatment approaches. The underlying goal is to help patients to live with and overcome disease. Mistletoe infusions are a means of accomplishing this goal. They are applied to stabilize disease, achieve responsiveness, induce fever, improve quality of life, and improve the tolerability of conventional cancer treatments. The doctors reported long-term disease stability and improvements in patients' general condition, vitality, strength, thermal comfort, appetite, sleep, pain from bone metastases, dyspnea in pulmonary lymphangitis carcinomatosa, fatigue, and cachexia; chemotherapy was better tolerated. Also patients' emotional and mental condition was reported to have improved.* Conclusion*. Individualized integrative cancer treatment including MT aims to help cancer patients to live well with their disease. Further research should investigate the reported observations.

## 1. Introduction

Mistletoe treatment (MT) is an essential part of integrative cancer care [1–5]. It is mostly used to improve quality of life (QoL), increase the tolerability of chemotherapy, and exert a possible benefit on tumor control and survival. Mistletoe extracts (MEs) contain a variety of biologically active compounds such as lectins, viscotoxins, oligo- and polysaccharides [6, 7], and triterpene acids [8]. They are cytotoxic, have strong apoptosis-inducing effects [9–11], enhance the cytotoxicity of anticancer drugs [12, 13], stimulate the immune system, possess DNA-stabilizing properties in mononuclear cells, and enhance endorphins* in vivo* [14, 15]. When injected into tumor-bearing animals, MEs inhibit and decrease tumor growth [14, 15]. In clinical practice, MEs are usually applied subcutaneously, starting with low doses that increase according to tolerability and local skin reactions or to lectin content. Various clinical studies have shown improvements in the QoL of cancer patients. A recent randomized controlled trial (RCT) found a highly statistical significant benefit of survival for patients with advanced pancreatic cancer [16]. Other studies have been inconsistent in this regard [17, 18]. Case reports and series have reported regressions of different tumor types after high-dose local applications of MEs [14, 19–25].

Despite the extensive body of scientific information, practicing physicians and experts have expressed the view that MEs are applied differently in clinical trials (i.e., in a highly standardized manner according to producer guidelines) than clinical reality, particularly when applied by highly experienced experts within an integrative setting.

ME treatment for cancer was largely developed by medical doctors, mostly within the context of anthroposophic medicine (AM), a healthcare approach that provides an integrative, multimodal, system-based cancer care [26]. Therapies and MT are individually applied, tailored to the specific medical condition and complaints of the patient; to his or her emotional, mental, spiritual, and social needs; and to his or her respective goals. Although global effectiveness studies have assessed individualized treatment applications [27–30], they do not provide detailed insights and covered only a small area. It remains unresolved whether such an individual way of applying MT brings about better results for patients, what those results are, and what characterizes such an individual treatment. It is of further interest to determine whether a study of concepts and internal evidence of highly experienced doctors can give rise to appropriate research and therapy development.

A qualitative study was conducted to gain insight into the concepts, goals, procedures, and observations associated with individualized cancer care; the long-term care of severely ill patients; the mental, emotional, and spiritual aspects involved; the sources of experts' knowledge and judgments; and whether the results convey clues for the further development of ME treatment. Interviews were conducted with highly experienced doctors. The study unearthed a wealth of information that is currently being published. One result was that physicians often stress the importance and potential of intravenous application of MEs, which differs from normal subcutaneous ME treatment and is a hardly known off-label type of intervention.

Therefore, the presented study aimed to focus on ME application as an intravenous infusion. It posed the following questions.

- Why are MEs applied as an intravenous infusion? What are the reasons, goals, and situations involved?
- How are the MEs applied? What are the procedures, differences, commonalities, and safety aspects?
- What do doctors observe? What are the benefits and risks?
- What are the concepts?
- How do these results compare with external evidence?

## 2. Methods

A qualitative guideline-based interview study was carried out with doctors highly experienced in integrative cancer care and MT in order to assess the doctors' concepts, procedures, experiences, and observations [31, 32]. The study was approved by the Ethics Committee of the University of Freiburg.

### 2.1. Sample

Participants were purposively sampled [33, 34]. The selection criteria included a spectrum of different therapy approaches, preparation methods, and doctors' medical specializations, treatment contexts (e.g., hospital or office-based practice and palliative or curative patients), ages, and countries. The doctors were contacted and received information about the study aims and interview durations and were asked to prepare oncological case examples.

### 2.2. Interviews

The interviews were conducted by two researchers (GK and MM) between 2009 and 2012. GK is a medical doctor and researcher and well known in the fields of integrative cancer care, AM, and MT. MM is a psychologist and researcher. All of the interviews were conducted face-to-face. Anonymity and high confidentiality were ensured, which enabled open communication. Most of the interviews took place in the work setting of the respective doctors. A few were conducted at the research institute or within the context of a congress. All interviews were carried out in a quiet undisturbed room. All doctors consented to digital audio recording except for one, whose interview consisted of field notes.

All of the interviews started with a warm-up question. The doctors then provided one or two case examples to give an uninfluenced account of their procedures, concepts, and observations. A guideline with interview questions was used as a checklist to ensure completeness of content and to follow up on certain topics [35]. It was constructed with input from the literature and external experts. Interview topics ranged from patient assessment to choosing mistletoe applications, preparations, host trees and doses, monitoring and adjusting treatments, time aspects, treatment goals, effectiveness, specific constellations, symptoms and complaints related to cancer disease, psychological and spiritual issues, additional therapies and influences, safety aspects, and new insights. The doctors were asked to concretize their answers and illustrate them using case examples. At the end of the interviews, each doctor was asked to fill in a short sheet with sociodemographic information. All of the interviews were transcribed by staff members of the research institute according to the approach suggested by Kuckartz [36]. The interviews were sent to the participants for member validation [33] and member checks were maintained throughout the different stages of the research process [32, 35]. After data collection from the 35 interviews was completed, it was assumed that no further relevant areas of information would be found [33].

### 2.3. Content Analysis

We used qualitative content analysis according to Mayring [31] and charting techniques of the thematic framework approach of Ritchie [33] to analyze the data. Data analysis was predominantly conducted by GK and MM using MAXQDA computer software [36] to manage the data, code and extract text passages, and search the text. Two other researchers took part in team meetings (HK, a researcher and medical doctor, and DF, a psychologist and experienced qualitative researcher). Analysis was done in close exchange between the researchers and its steps were documented.

We conducted two pilot interviews. We then discussed and specified the guidelines (e.g., put questions into the past tense to access observations) and conducted further interviews while starting initial analysis. In the first step, we read the interviews, noted the codes (open coding), and then combined the data with the codes from the interview guidelines [31]. Second, the domains for data extraction and further analyses were defined and extracted for each doctor (axial coding) [31]. In analyzing the doctors' intravenous applications, contents related to actual treatment procedures, QoL observations, tumor behavior, courses of disease, symptoms, psychological issues, and safety were extracted and their core meaning was summarized in a circular process to condense the given information. Words and phrases from the participants' own languages were used to stay as close to the original interview text as possible, and relevant quotes were kept alongside to ground the extracted themes in data [33]. Publications and literature referrals of the interviewees were included in charts to make what was said more explicit (explication) [31]. The charts were reviewed, discussed, and corrected by at least two researchers. The condensed information was merged into one chart covering all of the participants to find key themes (vertical analysis) [31], such as the doctors' reasons for applying ME infusions, proceedings, observations, and treatment concepts (selective coding).

All doctors were asked whether they would participate in publishing case reports. In the process, their patients' charts were checked and the patients and other attending physicians were contacted. Furthermore, several of the interviewees had published articles or books that served as additional sources of information for their reports.

The doctors received the interview transcripts and final analysis results before publication. The results contained anonymized codes instead of names so that the doctors could revise them. The codes were removed before publication.

For triangulation, the results were compared with external evidence. Clinical studies and trials on intravenous MT were systematically collected as reported elsewhere [14, 17, 37–39]. The inclusion criteria were (1) prospective or retrospective studies or trials, with or without control groups; (2) study populations made up of cancer patients; (3) intervention groups treated with intravenous infusions of MEs; (4) clinically relevant outcome parameters; (5) completion of the study; and (6) published or unpublished status. Studies were excluded if they only measured toxicity or tolerability (phase I trial), only measured immune stimulation, or were not conducted on cancer patients. There were no language restrictions. Earlier systematic reviews provided a quality assessment of these studies [14, 17, 37–39]. It was not possible to collect case reports in a completely systematic way, as they were usually published not in peer-reviewed journals but in other journals, books, brochures, and so forth. The case reports collected were therefore confined to those published by interviewees.

## 3. Results

Thirty-five interviews were conducted. Ten doctors could not be interviewed due to organizational problems (two), lack of response (four), illness (one), or unwillingness to present therapeutic intimacies in public (three). The interviews lasted between 100 and 297 (mean 171) minutes.

### 3.1. The Sample

**Figure 1:**
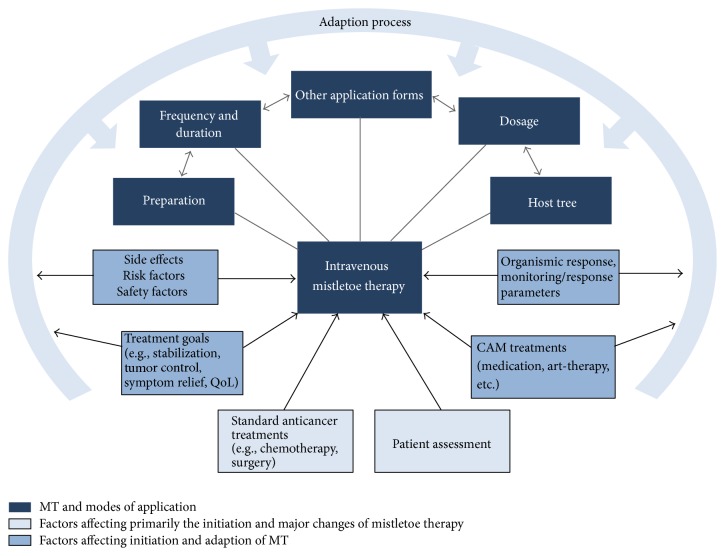
Intravenous mistletoe therapy: factors for choices and adaptions.

Table 1 shows the characteristics of the interviewed doctors. All of the doctors worked within an integrative treatment context, usually as part of a team of caregivers. They all worked in or collaborated with cancer centers or conventional experts (oncologists, surgeons, radiotherapists, etc.). Close collaboration within the team and other attending physicians was given significant consideration. Patient assessment was based on a precise diagnosis of tumor, stage, histology, symptoms, and relevant clinical, laboratory, and imaging evaluations. It included other relevant present and past conditions and complaints; functional, emotional, cognitive, social, and biographical issues; and goals and priorities to generate a whole “picture” of each patient [40]. Assessment was prioritized and a multimodal treatment approach tailored to the individual patient was pursued [40]. This also applied to MT and intravenous MT cases (Figure 1), which were individualized and adapted to the patient's diagnosis, condition, and evolving goals and later adjusted accordingly [40].

The doctors illustrated their reports with numerous case examples and some were published. Their arguments were usually critical and self-critical. Most of the doctors were careful or resistant to drawing any causal conclusions or generalizations. They reflected basic methodological principles, such as the difficulties involved in making assessments without control groups, and the presence of confounders and possible biases such as a “positivity bias,” in which case they would pass their own positive attitude onto their patients. They frequently referred to the results of clinical trials or other research. They were sometimes critical of mistletoe and its effects.

### 3.2. General Concepts

**Figure 2:**
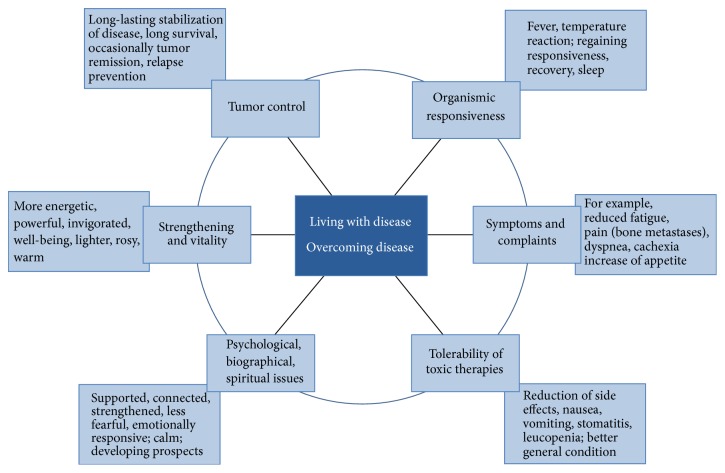
Concepts, goals, and observations associated with intravenous MT.

The interviewed doctors' global therapeutic concept underlying MT and AM cancer care was to enable patients to overcome disease, if possible, or to live* with* their disease and achieve a good condition in the long term even if the disease progressed. This global concept was associated with the following goals: tumor control and symptom relief; acceptance and good tolerability of standard cancer treatments; strengthening (i.e., physical, emotional, and mental strength; vitality; immune system); improving responsiveness and agility; gaining autonomy, also from the disease (acceptance, peace, perspectives, and being less bothered); the ability to recover (sleep, appetite, and restructuring); and the beneficial effects of overcoming a crisis, particularly fever (Figure 2).

### 3.3. Reasons for Applying Mistletoe Infusions

Doctors applied ME as intravenous infusion in the following situations, particularly to enhance the subcutaneous MT effect using larger doses without causing local reactions:

- Lack of response under subcutaneous treatment, when “*nothing changes anymore,*” to regenerate responsiveness.
- Stabilization and support in advanced, progressing, metastasizing diseases, when patients are in a critical situation or appear devitalized and when their “*strength flows out,*” to invigorate, strengthen, and consolidate patients (“*get ground under one's feet*”) and to stabilize the tumor situation.
- High-risk patients who have relapsed or are at risk of relapsing.
- To induce a fever reaction, stimulate the immune system, and raise the patient's temperature and feelings of warmth, to support recovery after adjuvant tumor treatment, and “*to structure the chaos again*.”
- Specific tumor situations such as gastric cancer with poor prognosis, prostate cancer and bone metastases, and advanced lung cancer and plasmacytoma not treated with chemotherapy.
- Improvement of QoL in general and in particular situations, such as pain, especially from bone metastases, fatigue, and dyspnea in lymphangitis carcinomatosa of the lung, and tumor cachexia, to improve the tolerability of chemotherapy.
- High-dose mistletoe induction in mistletoe-naïve patients, to elicit a fever response, start therapy with an intense high-dose concept, reduce tumor burden, decelerate tumor growth, stimulate the immune system before surgery or chemotherapy, and support patients in advanced, metastatic, and critical or palliative condition.

### 3.4. Side Effects and Safety Aspects

Doctors reported that hypersensitivity (pseudoallergic, rarely allergic) might occur more often under intravenous than subcutaneous treatment. Its symptoms, which include shivering, dyspnea and asthma, erythema, partly patchy or blistered, and cardiovascular reactions, are usually self-limited and occasionally require intervention. In addition to dose, this reaction was observed to be “*strictly dependent on dripping speed of the infusion […] When it is too fast, I can provoke a reaction in about everybody*” (general practitioner). To prevent these reactions, the drip rate of the infusion has to be slow and the patient must be instructed not to accelerate the infusion on his or her own, or the dose must be decreased and increased only carefully, the preparation changed (e.g., from Abnoba to Helixor), and primary high-dose MT must be confined to patients with no previous mistletoe contact. When the safety aspects were taken into regard these reactions were rarely observed and intravenous MT was safe in high doses. When patients continued to develop pseudoallergic reactions, infusion therapy was terminated.

Additional reported side effects included self-limited skin blistering under high-dose induction, cellulitis in a patient who had received an epithelial growth factor before MT, phlebitides in children, and flushes and resurgence of old injection sites. Fever induction and high-dose application could be very strenuous and tiring (“*they are worn out*”). Initial shivering, flu-like symptoms, and acute-phase reaction could occur. Strong emotional reactions were also induced (“*tears to flow*”). However, these reactions were of short duration, and the patients' well-being, strength, and mood subsequently improved tremendously, like “*a phoenix from the ashes*.”

Infusions are predominantly done in hospitals, special daybeds, or well-suited office-based practices under supervision of a physician and a skilled staff. Some of the doctors interviewed rarely used intravenous applications because external circumstances did not allow for it or the doctors found it too risky. An emergency case and medications to handle potential hypersensitivity should be available. Patients were informed about therapy, safety aspects, side effects, and their off-label status and signed informed consent sheets.

The doctors regarded high-dose fever-inducing infusions to be inappropriate for weak, end-stage patients and children with advanced disease and poor condition during chemotherapy or if the patients felt too worn out, had no emotional resources, or could not tolerate fever and shivering. Some doctors also considered patients suffering from brain tumors, tumor compression, liver metastases, or tumor fever as inappropriate for intravenous treatment.

### 3.5. Application, Preparations, Dose, and Time Aspects

Preparations (Box 1) were chosen depending on the therapeutic goal, the patient's situation, the working context, and personal preferences of the doctors. Doses depended on preparation, the patient's condition, and the therapeutic goal. Infusions continued for 1–4 hours. Treatment durations varied and could last for years, until death or until the patients improved.

### 3.6. Fever

The frequency, quality, and time course of fever responses differ with different ME preparations.

*Iscador* infusions repeatedly induce fever after about 2 hours, accompanied by influenza-like symptoms, chills, shivering, and feeling unwell. When high temperature is reached, the patients feel comfortable, appear rosy, and start to sweat. Fever goes up to about 39°C, lasts for approximately 3 hours, and then drops again, so that outpatients can go home on the same day. Slower infusions can cause higher fever responses.

*AbnobaViscum* infusions do not induce fever alone, but only when combined with a subcutaneous injection (which alone can also induce fever). Fever can usually be elicited only 3-4 times, and only in patients without prior MT. It rises 6–12 hours after subcutaneous injection, up to 39,5–39,8°C, lasts for about 8–24 hours, and is gone after 2-3 days. It is exhausting, sometimes accompanied by headaches, and patients have to recover before receiving the next dose.

Regarding* Helixor* some doctors did not observe a fever response, while others did, albeit inconsistently. The response may depend on very high dosages. Temperature rises after 3-4 hours up to 38–40°C or just 0.5–1°C, measurable only with appropriate instruments. The fever is easier to endure, and patients feel more relaxed and well and may not notice it at all.

Infusions are sometimes combined with whole-body hyperthermia to prolong and intensify the fever period. Fever can rise up to 41.8°C and patients may be exhausted for 2 days. Patients are supported to better handle the fever, increase their well-being, and relieve the side effects.

### 3.7. Observations of QoL

The doctors described that patients experience an improved QoL and well-being under intravenous mistletoe infusion: patients regularly become “*more powerful*” and “*feel lighter*.” When the patients struggle with increasing weakness in advanced stages, they feel more energetic, dynamic, stronger, and “*invigorated*” with mistletoe infusions and can stay in a good condition for a long time, despite their progressive disease. Patients experience improved inner strength in addition to physical condition and fitness. Their condition becomes stable and “*recovery is activated*.” The patients become warmer, sleep better, have better appetites, start to eat again, and shift from catabolism to anabolism. Their skin becomes rosy. These effects were described as intense and extensive.“*It is a wonderful therapy. I would do it myself and also give exactly that to my mother*” (gastroenterologist). “*After 5 days the patient reports: I feel much more powerful, I can get up again, I can go for walks twice as long as before. […] If somebody says, ‘I can only walk three steps from my bed into the kitchen and then I'm tired', then I can expect that they are more mobile again after one week of inpatient treatment and that they can walk again without support to a certain degree. If they say, ‘I can only walk with a walking aid,' then I want to see that they eventually can at least walk again with crutches*” (internist).

These improvements are reported for about 50% to 80% of patients and about 20% to 30% of children and to be repeatable. Pausing intravenous infusions, on the other side, can lead to a deterioration of the patients' condition. In terms of care of dying patients, some of the doctors considered infusions as ineffective; others reported that well-being still improves and that “*calm and light [comes] into the situation*” (oncologist).

Pain caused by bone metastases was reported to improve reliably and substantially. The pain does not subside all at once and might even increase for a short time, but it resolves after the third or fourth infusion. Connected functions might also improve. For instance, patients no longer require walking aids. Prior associated freezing and chilling give way to a pleasant feeling of warmth after infusions. It was observed that X-rays showed sclerosis of the metastases (with osteolytic destabilizing metastases always receiving radiotherapy). Visceral pain does not or only modestly improve in a similar way, and opiates could not be replaced. Rather, the emaciation experienced through exhausting pain is relieved.

Fatigue, such as chemotherapy-associated fatigue, was described to also improve substantially and frequently within 2–4 weeks. This necessitates two to three infusions per week.

An improvement of QoL during chemotherapy was reported in about 50–80% of patients receiving a mistletoe infusion. General tolerability and the vitality and mood of the patients increase, and levels of asthenia, fatigue, mucositis, nausea, vomiting, and leucopenia decrease. Dose adaptions and premature terminations of chemotherapy become less likely.

The doctors reported that the emotional and mental levels of the patients stabilize and strengthen and that their moods improve. Patients become emotionally responsive, find inner peace and calmness, are less anxious, feel at ease (“*secure*”), feel confident, and develop prospects for the future (“*to have perspectives and will power*” [gastroenterologist]). The doctors used the following terms to describe the changes: “*emotional coat of warmth*,” “*sun*,” “*light*,” “*brightness*,” “*rest in*,” and “*connect with themselves*.”

### 3.8. Observations in regard to Tumor Control

The doctors indicated that tumor control was very difficult to assess in individual patients. However, many of the doctors illustrated via numerous case examples that high-dose treatments with intravenous infusions were followed by a long-lasting stabilization of the disease and a much longer-term survival than predicted, with good condition and QoL (Box 2). For instance, patients with peritoneal disseminated ovarian cancer lived for years under stable conditions with repeated intravenous MT (Helixor) (“*They actually should have already died a long time ago and they are still alive*” [general practitioner]). Sometimes the condition improved after changing the mistletoe preparation or host trees or combining it with other remedies (Box 3).

Relevant tumor reduction with infusions was not expected and not observed by most of the doctors. Still, some physicians did observe durable tumor remissions with intravenous infusions, mostly in combination with other application forms (Box 4), which seemed to augment the tumor responses. For instance, long-lasting tumor remissions were achieved in Merkel cell carcinoma, breast cancer, primary cutaneous B-cell lymphoma (which have also been published [19, 20, 24]), head and neck cancers, and others. One doctor perceived intravenous MT as a “*turning point*” in courses with recurrent relapses (“*There are many examples of this kind where you have the impression that again it was a turning point*” [general practitioner]). However, the doctors also indicated that a clear differentiation from the spontaneous course of disease was not often possible.

### 3.9. External Evidence

Treatment of cancer patients with intravenous infusions with MEs has been investigated in four RCTs, one matched-pair study (see Table 2), and ten retrospective studies (see Table 3). Their methodological quality has been assessed elsewhere [14, 17, 37–39]. Three of the RCTs investigated the influence of MT infusion on tolerability of chemotherapy [41–43]. The fourth RCT [44] and matched-pair study [45] investigated its influence on surgery-induced suppression of the granulocyte function and NK-cell activity. Two of the studies investigated just a single infusion [44, 45]. One RCT also assessed survival [42]. The trials had small sample sizes and some lacked detailed information. The retrospective studies assessed the application of MT infusions in everyday practice, predominantly in patients with advanced, inoperable, and recurrent disease, but also partly in patients subsequent to surgery or radiotherapy. Most of the studies were published in the 1940s and 1950s, had methodological weaknesses, and investigated preparations no longer used in cancer therapy.

In terms of QoL, most of the trials reported an improved tolerability of chemotherapy, fewer side effects, a better general condition, and decreased anxiety. The retrospective studies mostly investigated patients with advanced metastatic disease. They described an improved subjective and general condition following intravenous MT; improved mental states; increases in well-being and initiative; a reduction of or freedom from symptoms despite disease progression; reductions of pain and fatigue; increases in weight, appetite, and physical strength; and better performance. Some of the studies also reported tumor remissions, partly in combination with intratumoral application and, in one study, with radiotherapy. The reported side effects were short-term fever and flu-like symptoms.

Studies investigating the safety of the intravenous application of MEs or recombinant mistletoe lectins found good tolerability and no toxicity [46–51]. One recent observational study assessed 475 cancer patients who had received 6,028 intravenous ME applications. Twenty-two patients had reported 32 adverse drug reactions (ADRs) of mild or moderate severity. ADRs were more frequent in early rather than advanced stages. ADRs were less frequent in intravenous than in subcutaneous application [52].

Several case reports on the intravenous infusion of MEs have been published, including some by the doctors interviewed in this study, and mostly exhibited a good reporting quality in accordance with the current CARE guidelines [53]. These cases mostly described tumor remissions and improvements of general condition during intravenous applications that were often combined with subcutaneous application and, if possible, intratumoral mistletoe intervention [19, 20, 54, 55].

## 4. Discussion

Doctors use individually applied intravenous MT to stabilize disease, improve QoL and general condition, strengthen patients, support tumor control, and relieve symptoms. The application is individually adapted. In a wider context the general therapeutic goal is to strengthen the patients' whole constitution, including physical, vegetative, emotional, spiritual, and social dimensions, and to enable them to overcome or live with their disease and improve their physical, emotional, and mental condition despite life-threatening or progressing disease and potentially harmful treatments. Inducing fever is considered important, based on the conceptual background of AM and on data about the restorative and preventive function of high feverous infections and the historic success of fever therapy with bacterial toxins [19, 20, 56, 57]. The therapeutic concepts are based on a holistic understanding of the human organism [26, 58].

The different therapeutic components are considered to comprise a* therapeutic system* that synergizes effects and thus enhances the chances of health improvement [26, 59–61]. Cancer disease and treatment are understood within the holistic paradigm [62, 63]. Great emphasis is also put on the active participation, autonomy, and self-responsibility of the patient [26, 38].

The therapeutic goals, concepts, and observations voiced by the doctors interviewed in this study are consistent with the reasons given by patients for consulting AM doctors. In addition to having their disease and symptoms treated, patients seek to strengthen their physical constitution and immune systems to better cope with the side effects of conventional treatments and improve their chances of being cured. They also seek a holistic approach (integration of psychic, spiritual, and biographical issues) and want to take greater responsibility for themselves and actively participate in treatment [30, 38, 64, 65].

The therapeutic goals, concepts, and observations identified in this study closely matched the problems, concerns, and needs of cancer patients in general. Patients often feel weak or tired, lack energy, or suffer from fatigue [66, 67]. Persistent chill and feeling too cold are underrecognized and underexplored forms of patient distress [68] that are presumably relevant for tumor control [69]. Disturbed sleep, pain, impaired taste or appetite, anorexia, and depression are additional problems [66, 67, 70, 71]. On the emotional level, substantial emotional distress—the sixth vital sign [72]—can be induced by diagnosis and treatment [73, 74]. Being younger and more educated increases a patient's risk of distress [75]. The ability to have a normal life without being too restricted by symptoms and to feel autonomous and in control of situations connected to one's personal life and treatment situation is essential [76, 77]. Functional limitations such as not being able to continue usual routines or daily life tasks and an inability to carry out important roles together with existential concerns are sources of suffering, particularly for palliative patients [75]. The needs of these patients often stay unmet and are underrepresented in the markers of good conventional oncology care, at least in Germany [78]. Therefore, a systematic approach toward individualized integrative care can complement conventional cancer care in a meaningful way.

The results from our interviews are consistent with the results from clinical mistletoe studies that have reported an improved general condition and mental state, decreased symptoms in patients with advanced disease, or improved tolerability of chemotherapy (see Tables 2 and 3). Other qualitative studies interviewing cancer patients about their experiences with MT or AM have reported similar issues: stronger vitality, enhanced autonomy, increased hope, better disease acceptance, and personal achievements such as new prospects, professional life changes, capacity to make own decisions and establish priorities, and improved self-confidence and strength [79–82]. Case reports have described condition stabilization or tumor remission, mostly under combined intravenous and subcutaneous and intratumoral ME application [19, 20, 54, 55].

Compared with the literature, this study illustrates a larger range of observations related to vitality, regaining strength and well-being, and condition stabilization and covers observation periods that last up to many years and decades. The safety aspects reported by the interviewed doctors provided more details than clinical studies [46–52].

### 4.1. Strengths and Weaknesses

The main strength of this study is the richness of information, arising directly from everyday clinical practice and from doctors who took care of their patients, often over years or even decades. Therefore, this study provides information about what may be pursued and possibly achieved in patients who are constrained by a serious life-threatening or life-limiting disease and are often suffering to a great extent. Additional strengths include the range of participants (achieved through purposive sampling), reflecting different specializations, countries, settings, and ages among other characteristics; the extensive interviews (up to 5 hours in duration); the amount of information gathered about complex therapy systems and treatment processes; the trusting and open atmosphere established through confidentiality; and the reputation of the researchers.

This study also has limitations. First, it presents only the views of doctors. The perspectives of patients were not evaluated and the treatment process was not directly observed. Both would have been important complements to the doctors' reports. In principle, however, other qualitative studies interviewing patients and patient surveys have reported similar outcomes. Patients judged AM cancer care in an AM hospital as particularly positive with regard to emotional effects, quality of human relations, and cognitive-spiritual effects, but also with regard to the effects on tumors and the body, mostly with reference to patients' recovery and general improvement. Their compliance with AM was high [27, 28]. In a British study, patients gave favorable acknowledgment of the time given to consultations, the quality of those consultations, the thoroughness involved in exploring medical and biographical histories, the combined conventional/AM approach, good and dialogue-like communication, care and personal encouragement, the holistic nature of the patient-centered approach, the benefits of individually tailored treatment, the facilitation of personal learning and development, and their personal involvement in the management of their illnesses [82]. Furthermore, patients' satisfaction with AM care has generally been high and their therapeutic expectations were fulfilled [30, 38, 83–85].

Other limitations include the confounding element of the integrative treatment setting which, as the doctors pointed out, impeded causal attributions to MT in most cases. Furthermore, our doctors, given their high levels of expertise in complementary and conventional cancer care and associated devices, may not be representative of the average caregiver. Many of the doctors had positive and empathic attitudes which possibly provided further additional support [86, 87]. However, although the doctors were careful when drawing causal conclusions and discussing the variety of confounders, they were certain about some specific therapeutic benefits of the intravenous application of MEs and made clear safety recommendations. Therefore, these benefits may be achieved in other therapeutic contexts as well and their further investigation is worthwhile. However, clear generalizations and the frequency of therapeutic effects are beyond the methods of this study.

Further research questions emerging from our results should be investigated in future clinical studies, including the influence of intravenous applications of MEs on (1) increasing weakness in progressive disease; (2) cancer-related fatigue; (3) pain caused by bone metastases; (4) tolerability of chemotherapy; (5) dyspnea in lymphangitis carcinomatosa of the lung; (6) tumor cachexia; (7) disease stabilization; and (8) tumor recurrence in high-risk patients. Secondary outcomes should include tumor control and patient survival, if possible. Furthermore, the effects and improvement of thermal comfort should be assessed in addition to functional abilities and issues of inner and outer autonomy. Close attention must be paid to the described individualization of treatment and safety aspects. A comparison of individualized treatment with standardized application or placebo administration would be a warranted treatment objective for later trials. If some of the presented observations are validated or replicated in clinical trials, they may contribute to the healthcare of cancer patients and help to relieve their suffering.

## 5. Conclusions

Individualized integrative cancer treatment including MT aims to help cancer patients to live well with their disease in many ways. According to the experiences of interviewed doctors, intravenous MT may particularly support patients in advanced stages and help stabilize and improve QoL and meet important needs and distresses of patients and help to positively affect a patient's tumor situation. Further research should investigate the reported observations.

## Figures and Tables

**Box 1:**
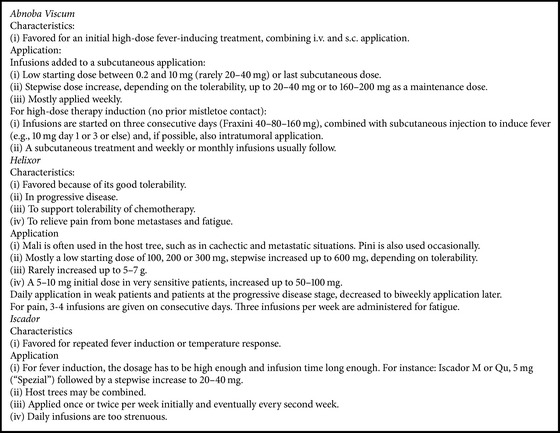
Examples of how the doctors applied infusions with different preparations.

**Box 2:**
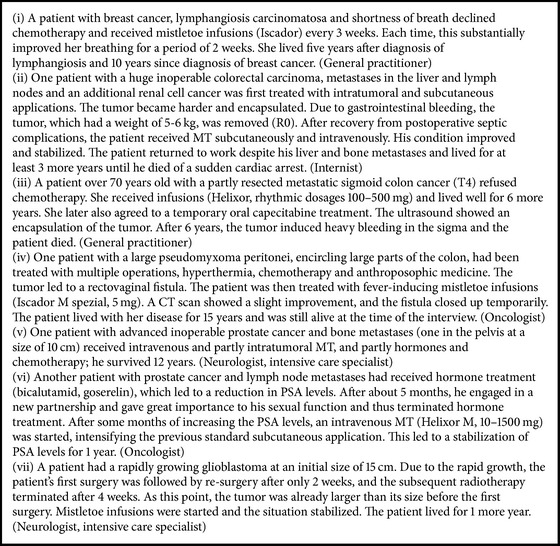
Case illustrations of favorite course of disease under MT infusions, as reported by interviewed doctors.

**Box 3:**
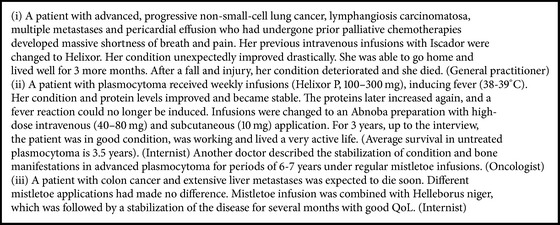
Case illustrations of favorite course of disease under infusions with changing ME preparations or combinations, as presented by interviewed doctors.

**Box 4:**
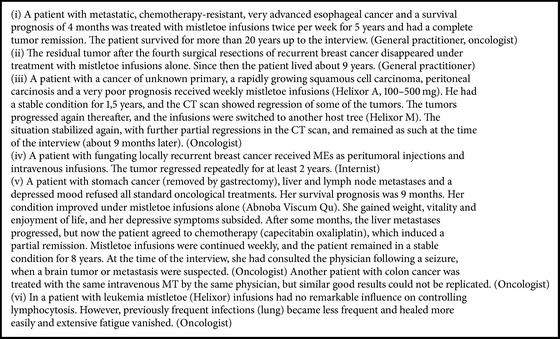
Case illustrations regarding tumor response and favorite course of disease under MT infusions.

**Table 1:** Sample characteristics: doctors using MT and integrative cancer care.

	Number	Years
		Median (range)
Doctors	35	
Men	30	
Women	5	
Age (years)		55 (40–84)
Specialty of doctor^**∗**^		
Oncology, hematology	8	
Internal medicine, pulmonology, or gastroenterology	17	
General practitioner	12	
Pediatrician	3	
Gynecology	1	
Neurology	1	
Research doctor	1	
Work experience as a physician		26 (11–57)
Cancer patients treated with ME/year: median (range)	270 (13–1,000)	
Using intravenous ME application	29	
Regularly	3	
Rarely	6	
Setting		
Hospital or outpatient clinic	21	
Resident doctor	14	
Working in or collaborating with cancer centers	35	
Country of workplace		
Germany	22	
Switzerland	6	
England, France, Sweden, Italy, Czech Republic, Egypt, Peru	1 from each country	

^**∗**^Some doctors had several specialties and are mentioned twice.

**Table 2:** Randomized and nonrandomized controlled clinical trials on intravenous mistletoe treatment in cancer.

Author, year	Site	Stage	Intervention (evaluable patients)	Survival		Immune parameters	Quality of life
Büssing et al. 2008 [41]	Breast	No data	(i) (5-Fu) EC, Iscador (32) (ii) (5-Fu) EC (33)			Granulocyte function, lymphocytes: no difference	Reduction of EC-related side effects: nausea, constipation, pain, stomatitis.^*∗*^ EORTC C30, BR 23: no difference

Schink et al. 2007 [44]	Colon, rectum	II–IV	(i) Surgery, Iscador^§^ (11) (ii) Surgery (11)			Decreased surgery-induced suppression of NK-cell activity^*∗*^	

Büssing et al. 2005 [45]	Breast (suspected)		(i) Surgery, Iscador^§^ (47) (ii) Surgery (51)			Decreased surgery-induced suppression of granulocyte function^*∗*^	

Cazacu et al. 2003 [42]	Colon, rectum	Dukes C and D	(i) Surgery, 5-Fu, Isorel (29) (ii) Surgery, 5-Fu (21) (iii) Surgery (14)	Median | mean survival (months)	Dukes C | D		5-FU side effects (% of pat.) 0% 19% QoL: ↑, data not shown
					25^*∗*^| 17^*∗*^		
					18 | 7		
					17 | 15		

Heiny 1991 [43]	Breast	Progredient	(i) VEC, Eurixor (21) (ii) VEC, placebo (19)				QoL ↑^*∗*^, anxiety ↓^*∗*^, leucopenia ↓^*∗*^. No effect on thrombocytes

§: single infusion. EC: epirubicin, cyclophosphamide; 5-Fu: 5-fluorouracil; V: vindesine; NK-cells: natural killer cells; ↑: increase; ↓: decrease; ^*∗*^statistically significant superior compared with control group.

**Table 3:** Single-arm retrospective studies of intravenous mistletoe treatment in cancer.

Author, year	Preparation	Cotherapy^i^	Tumor site^ii^	Tumor behaviour	*n* ^iii^	Quality of life
Wolf et al. 1994 [88]	Isorel		Diverse		25	Improved condition and mood, decreased pain and depression

Wolf 1987 [89]	Isorel		Diverse	Remissions	60	Improved subjective condition, appetite, digestion, weight gain

Brück 1950, 1954 [90, 91]	Plenosol	It	Diverse	Remissions	5	Improved general condition, well-being, symptom-free

Tosetti 1954 [92]	Plenosol	It, vitamins	Gynecologic		60	Improved general condition, weight gain

Rupp and Siegert 1952 [93]	Plenosol		Breast, cervix		50	Improved condition

Meythaler and Händel 1952 [94]	Plenosol		Diverse	Remissions	78	Improved condition, appetite, mood, physical strength, weight gain, decreased fatigue

Stehberger 1951 [95]	Plenosol	RT	Diverse		~40	Improved condition, weight gain, able to work again

Röseler 1952 [96]	Plenosol	It, surgery	Breast, gynecologic	Remissions	68	Improved physical strength, symptom-free despite progressing, disseminated disease

Wasmuht 1944 [97]	Plenosol	It, RT	ENT	Remissions	21	

Kraft 1940 [98]	Plenosol	It	Diverse	Remissions	27 (50)	Improved general condition, appetite, able to work again

^i^It: intratumoral application of mistletoe extract; RT: radiotherapy; ^ii^mostly advanced, inoperable, and recurrent; ^iii^ *n*: number of patients.
